# Associations between serum vitamin D status and the cardiometabolic profile of patients with obstructive sleep apnea

**DOI:** 10.1007/s42000-023-00456-4

**Published:** 2023-06-15

**Authors:** Michael Georgoulis, Meropi D. Kontogianni, Ioanna Kechribari, Roxane Tenta, Elizabeth Fragopoulou, Kallirroi Lamprou, Eleni Perraki, Emmanouil Vagiakis, Nikos Yiannakouris

**Affiliations:** 1 Department of Nutrition and Dietetics, School of Health Sciences and Education, Harokopio University of Athens, 70 El. Venizelou Str, 17676 Athens, Greece; 2 Center of Sleep Disorders, Evangelismos General Hospital, 1st Department of Critical Care and Pulmonary Services, Medical School, National and Kapodistrian University of Athens, 10676 Athens, Greece

**Keywords:** Sleep apnea, 25-Hydroxyvitamin D, Vitamin D deficiency, Metabolic syndrome, Obesity, Insulin resistance, Inflammation, Oxidative stress

## Abstract

**Purpose:**

Obstructive sleep apnea (OSA) and the metabolic syndrome (MetS) frequently coexist. Low serum vitamin D has been positively associated with OSA presence and severity; however, data on its link to cardiometabolic features in patients with OSA remain scarce. We aimed to assess serum 25-hydroxyvitamin D [25(OH)D] and explore its association with cardiometabolic parameters in OSA.

**Methods:**

This was a cross-sectional study among 262 patients (49 ± 9 years old, 73% men) with polysomnography-diagnosed OSA. Participants were evaluated in terms of anthropometric indices, lifestyle habits, blood pressure, biochemical, plasma inflammatory and urinary oxidative stress markers, and the presence of MetS. Serum 25(OH)D was assessed by chemiluminescence, and vitamin D deficiency (VDD) was defined as 25(OH)D < 20 ng/mL.

**Results:**

Median (1^st^, 3^rd^ quartile) serum 25(OH)D levels were 17.7 (13.4, 22.9) ng/mL and 63% of participants had VDD. Serum 25(OH)D correlated negatively with body mass index (BMI), homeostasis model of assessment of insulin resistance (HOMA-IR), total cholesterol, low-density lipoprotein cholesterol, triglycerides, high-sensitivity C-reactive protein (hsCRP), and urinary oxidized guanine species (oxG), and positively with high-density lipoprotein cholesterol (all *P* < 0.050). In logistic regression analysis, serum 25(OH)D was associated with lower odds of MetS [odds ratio (95% confidence interval): 0.94 (0.90–0.98)], after adjustment for age, sex, season of blood sampling, Mediterranean diet score, physical activity, smoking, apnea–hypopnea index, HOMA-IR, hsCRP, and oxG. In the same multivariate model, VDD was associated with ~ twofold greater odds of MetS [2.39 (1.15, 4.97)].

**Conclusion:**

VDD is highly prevalent and is associated with a detrimental cardiometabolic profile among patients with OSA.

**Supplementary Information:**

The online version contains supplementary material available at 10.1007/s42000-023-00456-4.

## Introduction

Obstructive sleep apnea (OSA) is a chronic sleep disorder characterized by recurrent partial or complete pauses of breathing during sleep, due to the obstruction of upper airways, which lead to intermittent hypoxia and sleep fragmentation [1]. Beyond its adverse impact on sleep duration, architecture, and quality, OSA also leads to a significant impairment of functionality, cognitive function, and quality of life [2], and most importantly to a significant burden on the cardiovascular system which is exposed to a vicious cycle of hemodynamic, oxidative, and inflammatory disturbances during sleep-disordered breathing [3]. In this context, OSA has been tightly linked to the metabolic syndrome (MetS) and its components, namely, abdominal obesity, impaired fasting glucose, dyslipidemia, and hypertension; these two pathological entities frequently coexist in a bidirectional relationship and combinedly lead to increased cardiometabolic morbidity [4]. Therefore, OSA, being more than just a sleep-related respiratory disorder, is currently recognized as a disease of cardiometabolic nature, and health organizations, such as the European Heart Association and the American Heart Association, classify OSA as a modifiable risk factor for the development of cardiovascular disease [5, 6].

Vitamin D represents a group of fat-soluble secosteroids with pleotropic effects that have been at the center of scientific and research interest since the beginning of the twentieth century. Besides its traditional role in regulating bone homeostasis and preventing metabolic bone disease, research over the last few decades has highlighted the ability of vitamin D to regulate gene expression by binding to its receptor in different cell types (e.g., immune, nervous, and cardiovascular cells) and thus affect the function of several systems of the human body [7]. The most well-studied extra-skeletal effects of vitamin D are related to inflammation, with evidence strongly indicating that vitamin D deficiency is implicated in the pathophysiology of inflammatory diseases and clinical conditions associated with chronic low-grade inflammation [8, 9]. Experimental and epidemiological studies have also reported a variety of other favorable cardiometabolic effects of vitamin D, including improved lipid metabolism, endothelial function, and insulin sensitivity, as well as anti-oxidant properties [10]. However, vitamin D deficiency remains highly prevalent worldwide, even in countries with long-established public health strategies for food vitamin D fortification and geographical locations with many hours of sunlight, such as the Mediterranean region [11]. Moreover, a number of previous epidemiological studies have explored the relationship between vitamin D status and OSA presence and severity, showing that serum vitamin D levels are lower in patients compared to controls and decrease as the disease progresses [12]; data on the association between serum vitamin D and the cardiometabolic profile of patients with OSA, however, remain sparse.

The aim of the present study was to assess serum vitamin D levels and the prevalence of vitamin D deficiency and explore their relationship with individual anthropometric, biochemical, inflammatory, and oxidative stress indices, as well as with the presence of the MetS and its components, in a sample of patients with polysomnography-diagnosed OSA.

## Methods

### Study protocol and population

**Fig. 1:**
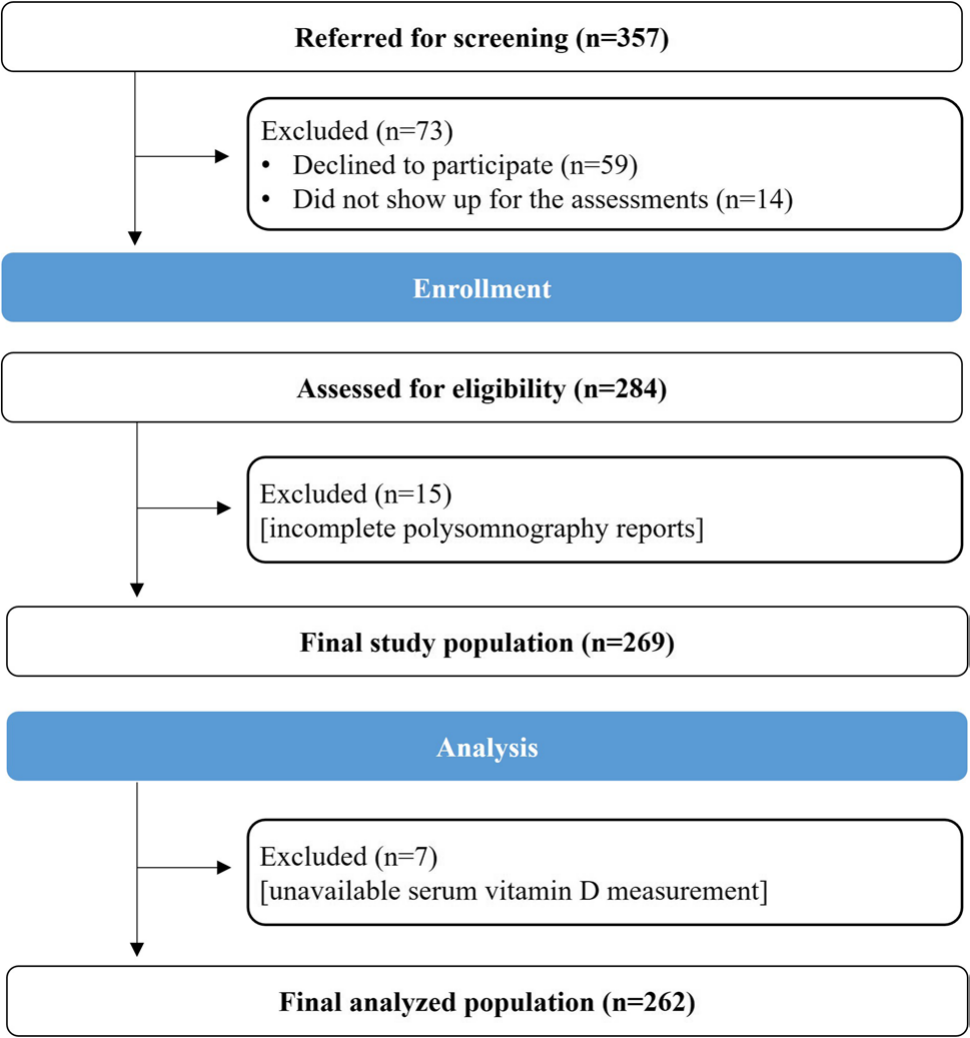
Study flowchart. From November 2014 to October 2018, 357 newly diagnosed patients with obstructive sleep apnea based on an attended overnight polysomnography were prospectively referred for screening by the research team. Of the 357 patients, 59 declined to participate and 14 did not show up for the assessments. In total, 284 patients were screened for eligibility. After excluding 15 patients who did not have complete polysomnography reports, 269 provided signed written consent and were enrolled. The working sample for current analyses consisted of 262 patients with available data on serum vitamin D status

Herein, we report data from a cross-sectional epidemiological study among adult patients with OSA. A detailed description of the study design, recruitment procedure, methodology, and population can be found in previous reports of our study group [13–15]. Briefly, the study sample consisted of adult males and females with a clinical suspicion of sleep-disordered breathing who were subjected to an overnight attended polysomnography (PSG) at the Center of Sleep Disorders of Evangelismos Hospital, Athens, Greece. OSA diagnosis was based on apnea–hypopnea index (AHI) values ≥ 5 events/h, and patients were further classified into those with mild (AHI: 5–14 events/h), moderate (AHI: 15–29 events/h), and severe (AHI ≥ 30 events/h) OSA [16]. A detailed interviewer-administered questionnaire was used to collect data on participants’ sociodemographic characteristics (i.e., date of birth, sex, total years of education, and mean annual income), individual and family medical history, medication use (type, dosage, frequency, and duration), habitual alcohol intake, and any recent attempt to change body weight or lifestyle habits in order to determine the study inclusion and exclusion criteria. Patients with sleep disorders other than OSA (e.g., central sleep apnea), other serious comorbidities (e.g., diabetes mellitus and cardiovascular disease), or recent hospitalization or surgery, those systematically receiving any medication affecting body weight and sleep (e.g., antidepressant and hypnotic drugs), alcohol abusers, and those who reported significant changes in body weight or lifestyle habits during the year preceding OSA diagnosis were excluded from the study. From November 2014 to October 2018, a total of 357 newly diagnosed patients with OSA were screened for eligibility; of these, 88 were excluded and the remaining 269 were enrolled after providing signed written consent. However, the working sample for the current analysis consisted of 262 patients with available data on serum vitamin D status (Fig. 1). The study protocol was approved by the Scientific Board of Evangelismos Hospital and by the Bioethics Committee of Harokopio University of Athens (approval number: 44/13–10-2014), and the study was conducted in accordance with the Declaration of Helsinki [17].

### Assessment of lifestyle habits

Participants’ habitual dietary intake over the last 6 months before PSG was assessed through a 76-item semi-quantitative food frequency questionnaire (FFQ), which has been validated in the Greek population [18]. The FFQ includes questions regarding the frequency of consumption (never/rarely, 1–3 times/month, 1–2 times/week, 3–6 times/week, 1 time/day, or ≥ 2 times/day) of all major food groups and individual foods/beverages typically consumed in Greece. Based on raw data, dietary intake was expressed in terms of daily food and food group consumption (servings/day), using serving sizes provided in the dietary guidelines for Greek adults [19]. Adherence to the Mediterranean diet was evaluated through the Mediterranean diet score (MedDietScore) [20], an a priori dietary index ranging from 0 to 55, with higher values indicating a greater level of adherence. Moreover, three non-consecutive 24-h dietary recalls, including 2 weekdays and 1 weekend day, were performed for each participant using the three-pass approach [21]. Each dietary recall was analyzed using Nutritionist Pro™ (Axxya Systems, Redmond, Washington, USA) to extract mean vitamin D intake (μg/day). The use of dietary supplements containing vitamin D (including multivitamins) was also recorded.

Participants’ physical activity level was assessed through the short version of the International Physical Activity Questionnaire (IPAQ), which has been validated in the Greek population [22]. The IPAQ provides information on involvement in walking, moderate-intensity activities (e.g., gardening, house cleaning, or recreational swimming) and high-intensity activities (e.g., running, aerobics or sports) during a typical week in terms of frequency (days/week) and duration (min/day), based on which total daily time of physical activity (min/day) was calculated for each participant. Moreover, each type of physical activity category was rated according to a mean metabolic equivalent of task (MET) value, i.e., 3.3 for walking, 4 for moderate-intensity activities, and 8 for high-intensity activities [23], and total weekly minutes of metabolic equivalent of tasks (METmin/week) were also calculated as a measure of exercise volume and intensity. Regarding smoking habits, current smokers were defined as participants who smoked ≥ 1 cigarette/day, former smokers as those who had ceased smoking ≥ 6 months prior to evaluation, and never smokers as those with no history of tobacco use.

### Assessment of cardiometabolic profile and vitamin D status

A detailed description of all anthropometric, clinical, and laboratory assessments can be found in previous reports of our study group [13–15, 24, 25] and in Online Resource 1. In brief, a standardized protocol was utilized to measure body weight and height, based on which body mass index (BMI) was calculated, as well as waist circumference (WC). Fasting blood (12-h) and first-morning urine samples were collected from patients and serum/plasma/urine aliquots were stored at − 80 °C until analysis. Fasting glucose, insulin, total cholesterol (TC), high-density lipoprotein cholesterol (HDLC), triglycerides (TG), alanine transferase (ALT), aspartate transferase (AST), and gamma-glutamyl transpeptidase (GGT) were measured in plasma samples, as previously described [13–15, 24, 25]. The Friedewald formula [26] was used to calculate low-density lipoprotein cholesterol (LDLC) and the homeostasis model of assessment of insulin resistance (HOMA-IR) was calculated according to Matthews et al. [27]. Plasma inflammatory indices, namely, high-sensitivity C-reactive protein (hsCRP) and adiponectin, and urinary oxidative stress markers, namely, 8-iso prostaglandin F2a (8-isoPGF2a) and oxidized guanine species (oxG), were measured using appropriate assays [13–15, 24, 25]. Systolic/diastolic blood pressure (SBP/DBP) was measured via an automatic device according to a standardized protocol. The presence of MetS was defined according to a joint interim statement of several health organizations published in 2009 [28].

Serum vitamin D status was assessed by measuring circulating levels of total 25-hydroxyvitamin D [25(OH)D], which is considered the most reliable measure of overall vitamin D status since it reflects vitamin D2 + D3 contributions from all sources [29]. Specifically, 25(OH)D was measured in ng/mL using a direct, competitive chemiluminescence immunoassay (LIAISON® 25 OH Vitamin D TOTAL Assay, Automated Analyzer Liaison Diasorin, ΜΑ-002/Α.8/04–04-2019, DiaSorin Inc., Stillwater, MN, USA). Vitamin D deficiency was defined as serum 25(OH)D levels < 20 ng/mL (< 50 nmol/L), vitamin D insufficiency as serum 25(OH)D levels between 20 and 30 ng/mL (50–75 mmol/L), and vitamin D sufficiency as serum 25(OH)D levels ≥ 30 ng/mL (≥ 75 nmol/L), according to the 2011 Endocrine Society guidelines for the evaluation, treatment, and prevention of vitamin D deficiency [30]. To account for the effect of season on serum vitamin D levels, a categorical variable indicative of the season of patients’ blood sample collection was created and used as a covariate in analyses (winter: December to February; spring: March to May; summer: June to August; and autumn: September to November). Serum vitamin D measurements were available in 262 out of the 269 enrolled patients, who composed the final sample for the present analyses (Fig. 1).

### Statistical analysis

We herein present secondary analyses of a cross-sectional epidemiological study with the primary aim of exploring associations between lifestyle habits and OSA severity. For the purpose of the present analyses, a post hoc power calculation was performed, using the G-Power software (version 3.1.9.7, Heinrich-Heine-Universität, Düsseldorf, Germany, http://www.gpower.hhu.de) to calculate the exact power achieved for performing multiple logistic regression analysis using vitamin D status as the independent variable and the presence of the MetS as the dependent one. The final analyzed sample size (*n* = 262) was sufficient to achieve a power of > 90% to detect a statistically significant effect size, allowing for a type-I error rate of 0.05. The Statistical Package for Social Sciences Version 23.0 (IBM SPSS Statistics for Windows, IBM Corp 2015, Armonk, NY, USA) was used for data analysis. Reported *p* values were based on two-sided tests and compared to a significance level of 5%.

Categorical variables are presented as absolute number (relative frequency), while numerical variables as mean ± standard deviation if normally distributed or as median (1^st^, 3^rd^ quartile) if skewed. The Kolmogorov–Smirnov test was used to evaluate the normality of numerical variables. Correlations between serum 25(OH)D levels and various cardiometabolic indices were tested through Spearman’s correlation coefficient (rho) and graphically illustrated through scatterplots. Differences between patients with MetS or vitamin D deficiency vs. those without were tested through Pearson’s chi-squared test for categorical variables, Student’s t-test for normally distributed numerical variables, or the Mann–Whitney *U* test for skewed numerical variables. Differences in serum 25(OH)D levels and in the prevalence of vitamin D deficiency between groups of patients with different number of MetS components were also tested through the Kruskal–Wallis test and Pearson’s chi-squared test, respectively, to evaluate the dose–response relationship between serum vitamin D status and MetS.

Multiple logistic regression analysis was lastly utilized to explore associations between serum vitamin D status, expressed as either 25(OH)D levels or vitamin D deficiency, and the presence of the MetS and its components, i.e., abdominal obesity, hyperglycemia, hypertriglyceridemia, low HDLC, and hypertension; results are presented as odds ratios (OR) and 95% confidence intervals (CI) for each 1 ng/mL increment of serum 25(OH)D or for patients with vitamin D deficiency, respectively. Three models were constructed to gradually adjust for confounders based on the study population characteristics [13–15], determinants of serum vitamin D status [31], and risk factors for MetS [32]: model 1, adjusted for age (years), sex (females, males) and the season of blood sampling (winter, spring, summer, and autumn) to control for seasonal variability in serum vitamin D; model 2, adjusted for variables in model 1 plus dietary and lifestyle habits, i.e., MedDietScore (range: 0–55), physical activity (min/day) and smoking status (never smokers–former smokers–current smokers); and model 3, adjusted for variables in model 2 plus AHI (events/hour) to adjust for OSA severity, as well as HOMA-IR, hsCRP (mg/L) and oxG (ng/mg Cr), to explore whether associations persist regardless of the degree of insulin resistance, inflammation, and oxidative stress. A sensitivity analysis was also performed among participants not receiving vitamin D supplements (*n* = 241).

## Results

**Fig. 2:**
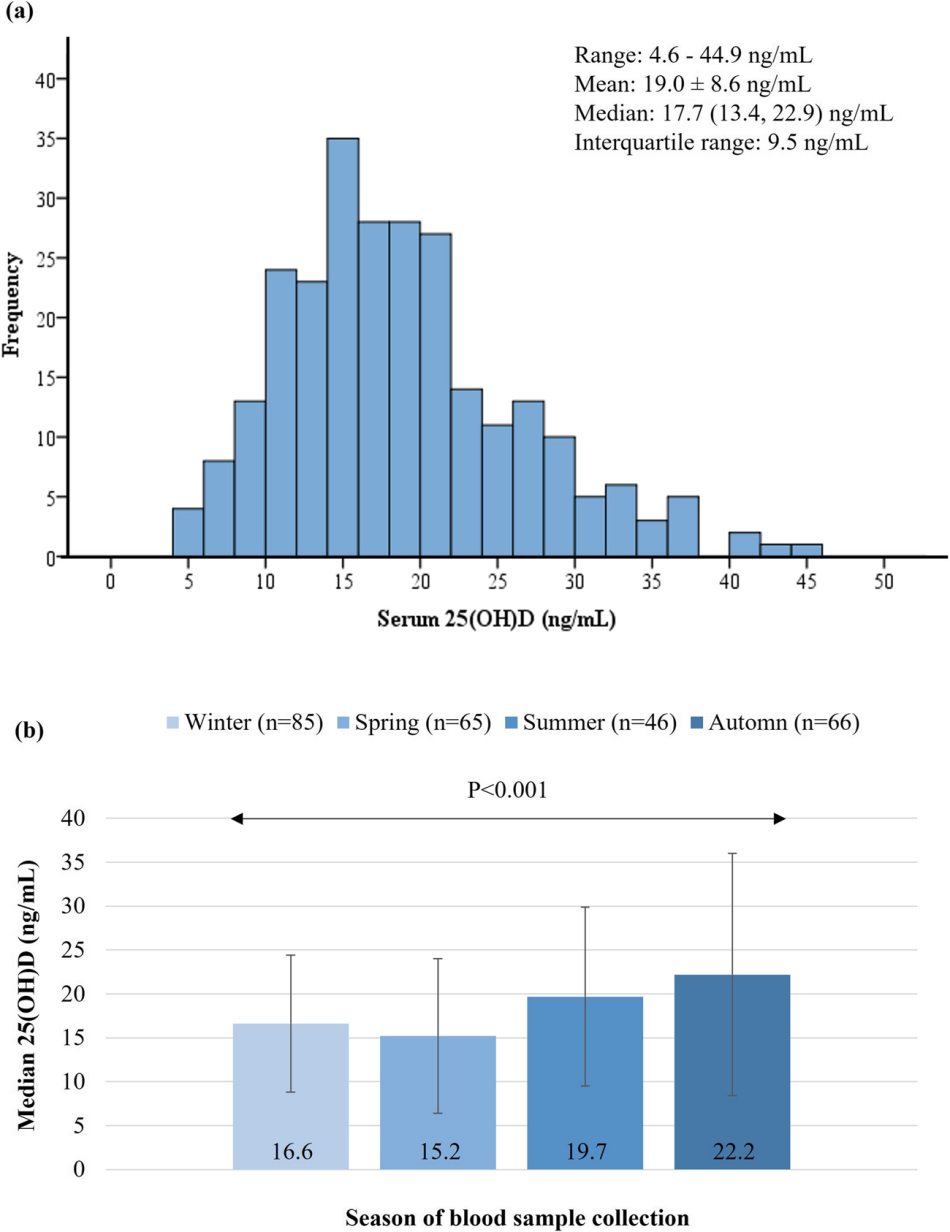
**a** Distribution of serum 25(OH)D values in the study population. **b** Serum vitamin D status according to the season of blood sampling (winter: December–February; spring: March–May; summer: June–August; autumn: September–November). Results on the *Y* axis correspond to median serum 25(OH)D levels and the (I) bars represent interquartile ranges. Between-group differences were tested through the Kruskal–Wallis test. Abbreviations: 25(OH)D, 25-hydroxyvitamin D

The descriptive characteristics of the study population are shown in Table 1. The sample consisted of 262 newly diagnosed patients with OSA (191 males and 71 females, male-to-female ratio: 2.7) with a mean age of 49 ± 9 years and a median (1^st^, 3^rd^ quartile) AHI of 46.5 (23.0, 80.0) events/hour (67.2% had severe OSA). Patients’ mean BMI was 35.2 ± 6.3 kg/m^2^ and 98.5% were overweight or obese (BMI ≥ 25 kg/m^2^). Patients reported a moderate adherence to the Mediterranean diet (mean MedDietScore: 32.3 ± 4.4), were mainly physically inactive (66.0% reported less than 30 min/day of any kind of physical activity), reported an average night-time sleep duration of 6.21 ± 1.60 h/day, and 33.2% were current smokers. Regarding vitamin D status, median (1^st^, 3^rd^ quartile), serum 25(OH)D levels were 17.7 (13.4, 22.9) ng/mL and varied significantly according to the season of blood sampling (a detailed distribution of serum 25(OH)D in the total study population and by season is presented in Fig. 2), and 63.0% of patients had vitamin D deficiency [< 20 ng/mL]. Median (1^st^, 3^rd^ quartile) dietary vitamin D intake was 1.70 (0.81, 3.26) μg/day, and 8.0% of participants reported using supplements containing vitamin D. The cumulative prevalence of MetS was 56.9%; the most frequent MetS components were abdominal obesity (83.6%) and hypertension (71.4%), followed by low HDLC (53.4%), hypertriglyceridemia (42.0%), and hyperglycemia (22.1%).

**Table 1:** Descriptive characteristics of the study population in total and according to the presence of the metabolic syndrome

	Total sample (*n* = 262)	With MetS (*n* = 149)	Without MetS (*n* = 113)	*P*^a^
Sociodemographic characteristics				
Age, years	49 ± 9	50 ± 10	48 ± 10	0.184
Males, *n* (%)	191 (72.9)	109 (73.2)	82 (72.6)	0.916
Educational level, *n* (%)^b^				
Low	21 (8.0)	13 (8.7)	8 (7.1)	0.537
Medium	122 (46.6)	71 (47.7)	51 (45.1)	
High	119 (45.4)	65 (43.6)	54 (47.8)	
Financial status, *n* (%)^c^				
Low	88 (33.6)	53 (35.6)	35 (31.0)	0.710
Medium	121 (46.2)	68 (45.6)	53 (46.9)	
High	53 (20.2)	28 (18.8)	25 (22.1)	
Anthropometric indices				
BMI, kg/m^2^	35.2 ± 6.3	36.4 ± 5.8	33.6 ± 6.7	< 0.001
BMI status, *n* (%)				
Normal weight	4 (1.5)	0 (0.0)	4 (3.5)	< 0.001
Overweight	56 (21.4)	18 (12.1)	38 (33.6)	
Obese	202 (77.1)	131 (87.9)	71 (62.8)	
WC, cm				
Males	118 ± 16	121 ± 13	114 ± 19	0.004
Females	110 ± 15	113 ± 15	105 ± 15	0.023
Lifestyle habits				
MedDietScore (0–55)	32.3 ± 4.4	32.6 ± 4.4	32.4 ± 4.5	0.354
Physical activity, min/day	14.6 (4.29, 38.6)	11.4 (0.00, 37.5)	17.1 (8.52, 40.3)	0.027
> 30 min/day, *n* (%)	89 (34.0)	48 (32.2)	41 (36.3)	0.491
Physical activity, MET-min/week	347 (100, 990)	264 (0.00, 990)	462 (181, 957)	0.029
Sleep duration, h/day	6.21 ± 1.60	6.30 ± 1.76	6.10 ± 1.36	0.334
Current smokers, *n* (%)	87 (33.2)	50 (33.6)	37 (32.7)	0.890
Ever smokers, *n* (%)	164 (62.6)	98 (65.8)	66 (58.4)	0.222
Polysomnographic characteristics				
AHI, events/h	46.5 (23.0, 80.0)	58.0 (27.0, 87.0)	39.0 (19.0, 74.0)	0.005
OSA severity, *n* (%)^d^				
Mild	27 (10.3)	11 (7.4)	16 (14.2)	0.039
Moderate	59 (22.5)	28 (18.8)	31 (27.4)	
Severe	176 (67.2)	110 (73.8)	66 (58.4)	
Cardiometabolic profile				
SBP, mm Hg	130 ± 15	133 ± 15	126 ± 13	< 0.001
DBP, mm Hg	85 ± 12	86 ± 11	82 ± 14	0.011
Glucose, mg/dL^e^	95 ± 18	98 ± 22	89 ± 8	< 0.001
Insulin, μU/mL^f^	14.6 (9.40, 22.5)	16.7 (12.4, 25.1)	11.4 (7.73, 18.1)	< 0.001
HOMA-IR	3.35 (2.04, 5.09)	4.05 (2.78, 5.91)	2.33 (1.68, 4.14)	< 0.001
TC, mg/dL^g^	188 ± 35	191 ± 39	185 ± 28	0.134
LDLC, mg/dL^g^	119 ± 30	120 ± 33	117 ± 24	0.426
HDLC, mg/dL^g^	42 ± 10	37 ± 8	48 ± 10	< 0.001
TG, mg/dL^h^	123 (89, 169)	157 (104, 212)	98 (72, 124)	< 0.001
ALT, U/L	20 (17, 25)	20 (16, 25)	20 (17, 24)	0.968
AST, U/L	24 (18, 34)	24 (19, 34)	23 (17, 33)	0.344
GGT, U/L	25 (18, 36)	25(18, 37)	24 (17, 34)	0.345
hsCRP, mg/L	2.71 (1.25, 5.30)	3.24 (1.43, 5.68)	2.24 (0.91, 4.36)	0.008
Adiponectin, μg/mL	3.60 (2.30, 5.76)	3.06 (2.07, 5.01)	4.32 (2.82, 6.42)	< 0.001
8-isoPGF2a, ng/mg Cr	0.57 (0.35, 0.98)	0.57 (0.34, 1.06)	0.56 (0.36, 0.85)	0.628
oxG, ng/mg Cr	64.5 (46.7, 93.6)	64.2 (47.7, 96.0)	65.1 (45.7, 91.7)	0.696
Vitamin D status				
25(OH)D, ng/mL^i^	17.7 (13.4, 22.9)	15.7 (11.8, 21.4)	19.4 (15.8, 25.3)	< 0.001
Serum vitamin D status, *n* (%)				
Deficiency	165 (63.0)	104 (69.8)	61 (54.0)	0.028
Insufficiency	73 (27.9)	35 (23.5)	38 (33.6)	
Sufficiency	24 (9.2)	10 (6.7)	14 (12.4)	
Vitamin D intake, μg/day^j^	1.70 (0.81, 3.26)	1.73 (0.72, 3.67)	1.61 (0.87, 2.74)	0.727
Use of vitamin D containing supplements, *n* (%)	21 (8.0)	7 (4.7)	14 (12.4)	0.023

Data are presented as absolute number (relative frequency) for categorical variables, mean ± standard deviation for normally distributed numerical variables and median (1^st^, 3^rd^ quartile) for skewed numerical variables.

AHI, apnea–hypopnea index; ALT, alanine transferase; AST, aspartate transferase; BMI, body mass index; Cr, creatinine; DBP, diastolic blood pressure; GGT, gamma-glutamyl transpeptidase; HDLC, high-density lipoprotein cholesterol; HOMA-IR, homeostasis model assessment of insulin resistance; hsCRP, high-sensitivity C-reactive protein; LDLC, low-density lipoprotein cholesterol; MedDietScore, Mediterranean diet score; MetS, metabolic syndrome; OSA, obstructive sleep apnea; oxG, oxidized guanine species; SaO2, oxygen saturation; SBP, systolic blood pressure; TC, total cholesterol; TG, triglycerides; WC, waist circumference; 8-isoPGF2a, 8-iso prostaglandin F2a; 25(OH)D, 25-hydroxyvitamin D.

^a^*P* value for the difference between groups as derived through Pearson’s chi-squared test for categorical variables, Student’s *t*-test for normally distributed numerical variables, or the Mann–Whitney *U* test for skewed numerical variables.

^b^Educational level was categorized as low, medium, and high if total years of education (primary, secondary, and tertiary) were < 6, 7–13, and ≥ 14, respectively.

^c^Financial status was categorized as low, medium, and high if mean annual income was < 10,000 €, 10,000–20,000 €, and > 20,000 €, respectively.

^d^OSA severity was classified as mild, moderate, and severe if AHI values were 5–14 events/h, 15–29 events/h, and ≥ 30 events/h, respectively.

^e^To convert glucose to mmol/L multiply mg/dL by 0.0555.

^f^To convert insulin to pmol/L multiply μIU/L by 6.00.

^g^To convert cholesterol to mmol/L multiply mg/dL by 0.02586.

^h^To convert TG to mmol/L multiply mg/dL by 0.01129.

^i^To convert serum 25(OH)D to nmol/L multiply ng/mL by 2.5

^j^To convert vitamin D intake to IU/day multiply μg/day by 40.

Comparisons in various sociodemographic, anthropometric, lifestyle, polysomnographic, and laboratory parameters between patients with MetS and those without are also presented in Table 1. No significant between-group differences were observed in sociodemographic data or lifestyle habits (all *P* > 0.050), apart from physical activity level, expressed either as min/day or as METmin/week, which was significantly lower in patients with MetS compared to those without (*P* = 0.027 and *P* = 0.029, respectively). A significant difference was also observed in polysomnographic parameters, with patients with MetS exhibiting higher AHI values (*P* = 0.005) and higher rates of severe OSA (*P* = 0.039). Regarding cardiometabolic indices, compared to MetS-free participants, those with MetS exhibited higher BMI, WC, obesity rates, SBP, DBP, fasting glucose, insulin, HOMA-IR, TG, and hsCRP, and lower HDLC and adiponectin (all *P* < 0.050). Dietary vitamin D intake was similar between patients with MetS and their MetS-free counterparts (*P* = 0.727), while more patients without MetS used dietary supplements containing vitamin D compared to those with MetS (*P* = 0.023). In addition, a significant difference was observed in serum vitamin D status, with patients with MetS exhibiting lower median (1^st^, 3^rd^ quartile) serum 25(OH)D levels and higher prevalence of vitamin D deficiency (< 20 ng/mL) compared to those without [15.7 (11.8, 21.4) ng/mL vs. 19.4 (15.8, 25.3) ng/mL, *P* < 0.001 and 69.8% vs. 54.0%, *P* = 0.028, respectively).

The cardiometabolic profile of the study population stratified by the presence of vitamin D deficiency is presented in Table 2. Compared to patients with vitamin D insufficiency or sufficiency, those with deficiency exhibited higher BMI (*P* < 0.001) and WC (*P* = 0.012 and *P* = 0.002 for males and females, respectively). Regarding individual biochemical, inflammatory, and oxidative stress indices, TC, LDLC, TG, AST, hsCRP, and oxG levels were higher, while HDLC levels were lower in subjects with vitamin D deficiency compared to those without (all *P* < 0.050). Patients with vitamin D deficiency also exhibited higher rates of abdominal obesity (*P* = 0.016), hypertriglyceridemia (*P* = 0.002), and low HDLC levels (*P* = 0 0.005), while the prevalence of hyperglycemia and hypertension was similar between the two groups (*P* = 0.165 and *P* = 0.345, respectively). The cumulative presence of MetS was 63.0% among participants with vitamin D deficiency and 46.4% among those without (*P* = 0.009).

**Table 2:** Cardiometabolic profile of the study population according to the presence of vitamin D deficiency [serum 25(OH)D < 20 ng/mL]

	With vitamin D deficiency (*n* = 165)	Without vitamin D deficiency (*n* = 97)	*P*^a^
Anthropometric indices			
BMI, kg/m^2^	36.4 ± 6.6	33.1 ± 5.3	< 0.001
BMI status, *n* (%)			0.072
Normal weight	3 (1.8)	1 (1.0)	
Overweight	28 (17.0)	28 (28.9)	
Obese	134 (81.2)	68 (70.1)	
WC, cm			
Males	121 ± 17	115 ± 13	0.012
Females	113 ± 15	101 ± 13	0.002
Blood pressure			
SBP, mm Hg	130 ± 14	132 ± 16	0.306
DBP, mm Hg	84 ± 13	85 ± 11	0.653
Glucose metabolism			
Glucose, mg/dL^b^	96 ± 20	92 ± 14	0.115
Insulin, μU/mL^c^	17.2 (12.1, 25.4)	10.5 (7.40, 16.7)	< 0.001
HOMA-IR	3.99 (2.70, 5.87)	2.21 (1.62, 2.21)	< 0.001
Lipidemic profile			
TC, mg/dL^d^	193 ± 37	181 ± 30	0.010
LDLC, mg/dL^d^	122 ± 31	114 ± 26	0.037
HDLC, mg/dL^d^	41 ± 10	44 ± 10	0.020
TG, mg/dL^e^	135 (95, 189)	103 (82, 143)	< 0.001
Liver enzymes			
ALT, U/L	20 (17, 26)	20 (16, 24)	0.921
AST, U/L	24 (19, 36)	23 (16, 30)	0.037
GGT, U/L	25 (18, 37)	26 (17, 34)	0.270
Inflammatory markers			
hsCRP, mg/L	3.32 (1.40, 5.75)	2.28 (0.91, 3.78)	0.006
Adiponectin, μg/mL	3.65 (2.36, 5.47)	3.57 (2.18, 6.39)	0.832
Oxidative stress markers			
8-isoPGF2a, ng/mg Cr	0.59 (0.32, 1.08)	0.57 (0.39, 0.83)	0.772
oxG, ng/mg Cr	68.0 (50.4, 103)	63.0 (40.3, 78.1)	0.010
Metabolic syndrome, *n* (%)^f^	104 (63.0)	45 (46.4)	0.009
Abdominal obesity, *n* (%)^g^	141 (85.5)	75 (77.3)	0.016
Hyperglycemia, *n* (%)^h^	41 (24.8)	17 (17.5)	0.165
Hypetriglyceridemia, *n* (%)^i^	81 (49.1)	29 (29.9)	0.002
Low HDLC, *n* (%)^j^	99 (60.0)	41 (42.3)	0.005
Hypertension, *n* (%)^k^	115 (69.7)	72 (74.2)	0.345

Data are presented as absolute number (relative frequency) for categorical variables, mean ± standard deviation for normally distributed numerical variables and median (1^st^, 3^rd^ quartile) for skewed numerical variables.

ALT, alanine transferase; AST, aspartate transferase; BMI, body mass index; Cr, creatinine; DBP, diastolic blood pressure; GGT, gamma-glutamyl transpeptidase; HDLC, high-density lipoprotein cholesterol; HOMA-IR, homeostasis model of assessment of insulin resistance; hsCRP, high-sensitivity C-reactive protein; LDLC, low-density lipoprotein cholesterol; MetS, metabolic syndrome; oxG, oxidized guanine species; SBP, systolic blood pressure; TC, total cholesterol; TG, triglycerides; WC, waist circumference; 8-isoPGF2a, 8-iso prostaglandin F2a; 25(OH)D, 25-hydroxyvitamin D.

^a^*P* value for the difference between groups as derived through Pearson’s chi-squared test for categorical variables, Student’s t-test for normally distributed numerical variables or the Mann–Whitney *U* test for skewed numerical variables.

^b^To convert glucose to mmol/L multiply mg/dL by 0.0555.

^c^To convert insulin to pmol/L multiply μIU/L by 6.00.

^d^To convert cholesterol to mmol/L multiply mg/dL by 0.02586.

^e^To convert TG to mmol/L multiply mg/dL by 0.01129.

^f^ According to the criteria proposed by Alberti et al. [28]

^g^WC values > 102 cm for males and > 88 cm for females.

^h^Fasting glucose levels ≥ 100 mg/dL (≥ 5.6 mmol/L) or reception of antidiabetic medication.

^i^TG levels ≥ 150 mg/dL (≥ 1.7 mmol/L) or reception of lipid-lowering medication.

^j^HDLC levels < 40 mg/dL (< 1.0 mmol/L) for males and < 50 mg/dL (< 1.3 mmol/L) for females, or reception of relevant medication.

^k^SBP ≥ 130 mm Hg or/and DBP ≥ 85 mm Hg, or reception of antihypertensive medication.

**Fig. 3:**
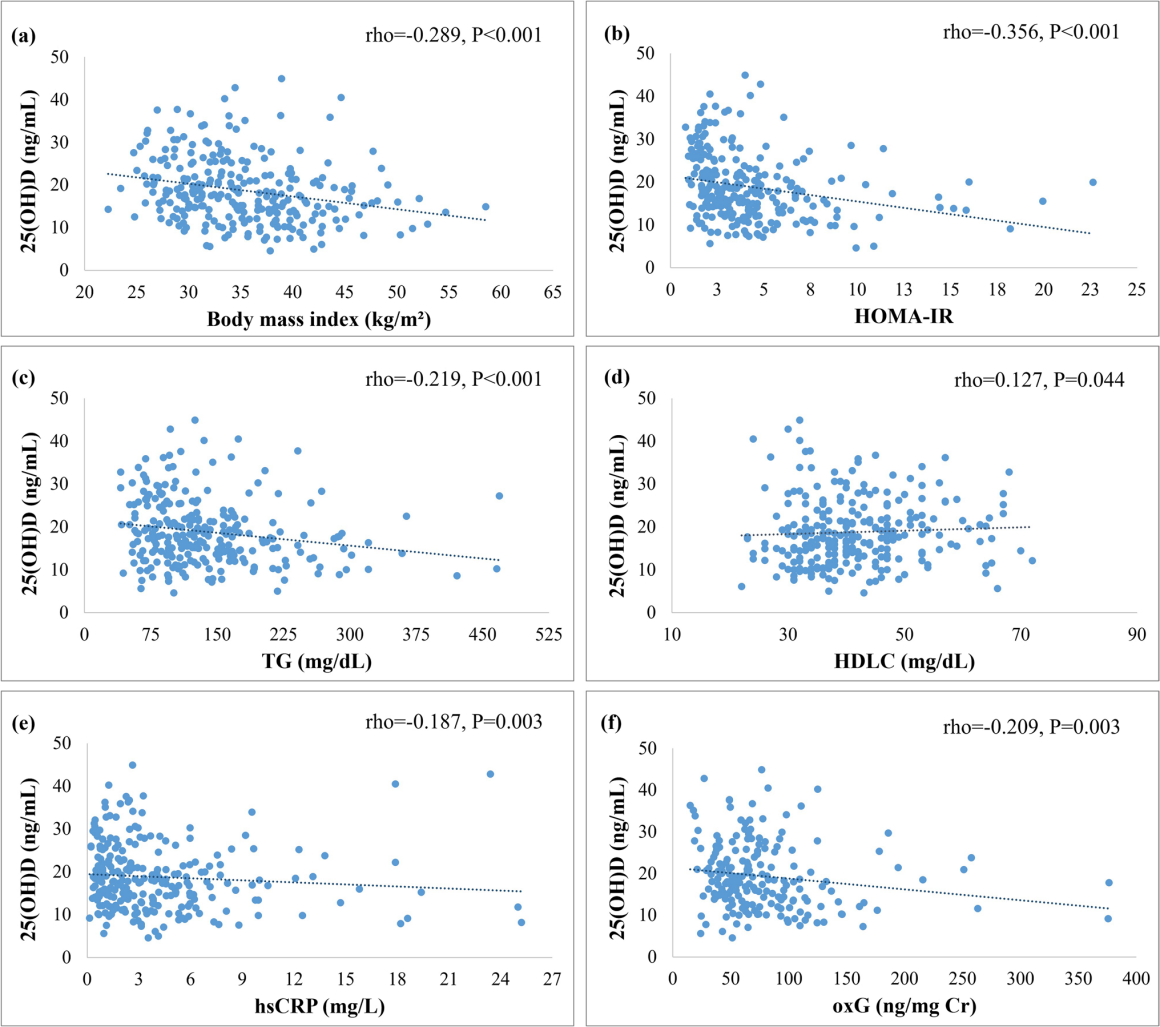
Scatterplots illustrating the association between serum 25(OH)D levels and **a** BMI, **b** HOMA-IR, **c** TG, **d** HDLC, **e** hsCRP, and **f** oxG. Dotted lines represent the linear regression curve (fit line) for the relationship between variables. Abbreviations: BMI, body mass index; HDLC, high-density lipoprotein cholesterol; HOMA-IR, homeostasis model of assessment of insulin resistance; hsCRP, high-sensitivity C reactive protein; oxG, oxidized guanine species; TG, triglycerides; 25(OH)D, 25-hydroxivitamin D

**Fig. 4:**
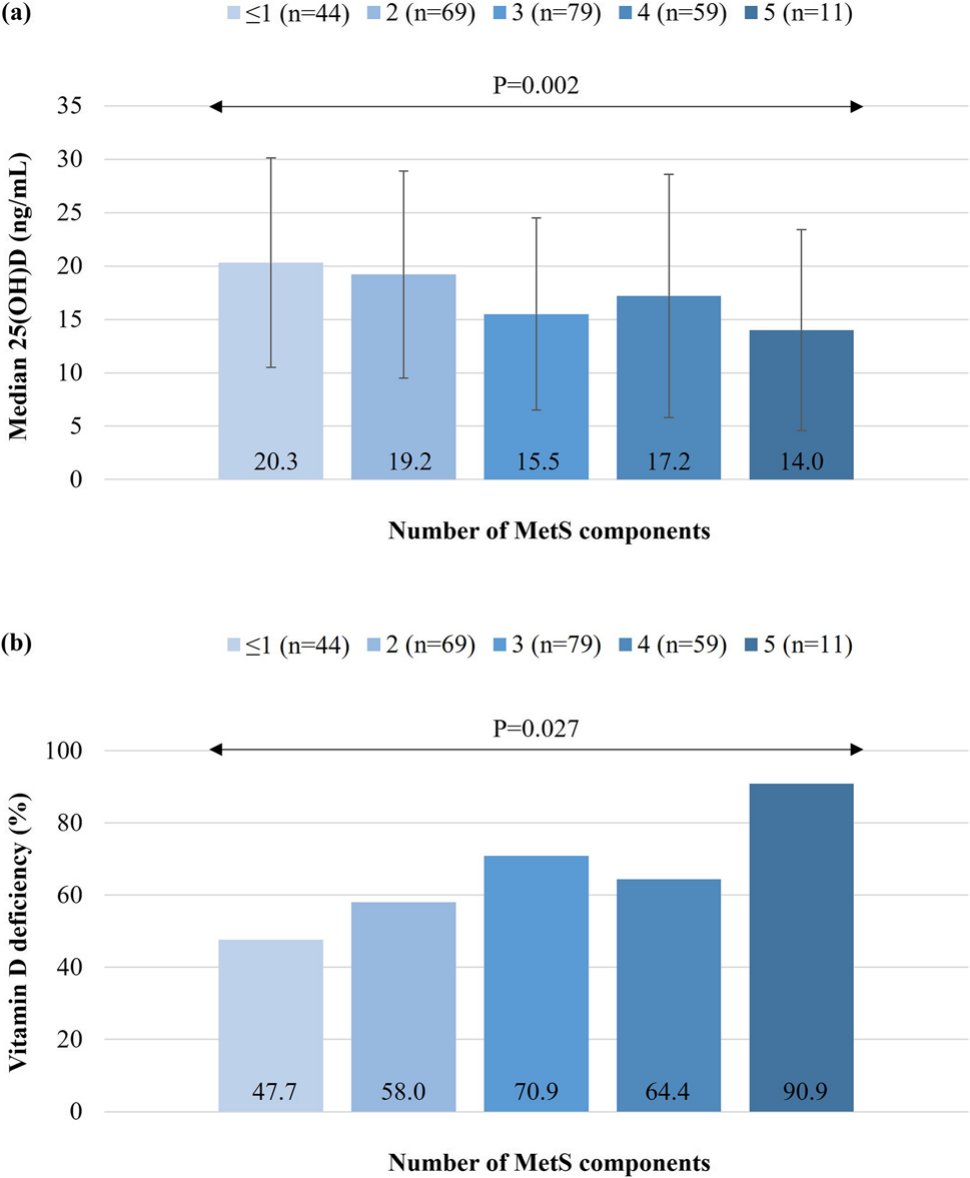
Association between vitamin D status and the number of MetS components. Since only four patients had no MetS components, the lower two categories, i.e., 0 and 1, were combined as ≤ 1. **a** Results on *Y* axis correspond to median serum 25(OH)D levels and the (I) bars represent interquartile ranges. Between-group differences were tested through the Kruskal–Wallis test. **b** Results on the *Y* axis correspond to the prevalence (%) of vitamin D deficiency [serum 25(OH)D levels < 20 ng/mL]. Between-group differences were tested through Pearson’s chi-squared test. Abbreviations: MetS, metabolic syndrome; 25(OH)D, 25-hydroxyvitamin D

In the total study sample, serum 25(OH)D levels were negatively correlated with BMI (rho =  − 0.289, *P* < 0.001), WC (rho =  − 0.221, *P* < 0.001), HOMA-IR (rho =  − 0.356, *P* < 0.001), TC (rho =  − 0.181, *P* = 0.004), LDLC (rho =  − 0.136, *P* = 0.030), TG (rho =  − 0.219, *P* < 0.001), hsCRP (rho =  − 0.187, *P* = 0.003), and oxG (rho =  − 0.209, *P* = 0.003) and positively correlated with HDLC (rho = 0.127, *P* = 0.044) (Fig. 3), whereas correlations between serum 25(OH)D levels and blood pressure, liver enzymes, plasma adiponectin, and urinary 8-isoPGF2a were not statistically significant (data not shown). A dose–response relationship was also observed between serum vitamin D status and the number of MetS components (Fig. 4). Specifically, median (1^st^, 3^rd^ quartile) serum 25(OH)D levels ranged from 20.3 (15.8, 25.7) ng/mL in patients with ≤ 1 MetS component to 14.0 (9.1, 18.5) ng/mL in patients with all five MetS components, and a significant between-group difference was observed (*P* = 0.002). A similar pattern was also evident for the prevalence of vitamin D deficiency (from 47.7% in patients with ≤ 1 MetS component to 90.9% in patients with all five MetS components, *P* = 0.027).

According to multiple logistic regression analysis (Table 3), each 1 ng/mL increase in serum 25(OH)D was associated with a 7% lower likelihood of MetS presence (OR: 0.93, 95% CI: 0.89–0.97), after adjusting for age, sex, and season of blood sample collection (model 1); this protective effect remained significant even after further adjustment for the MedDietScore, physical activity level, and smoking habits (OR: 0.93, 95% CI: 0.89–0.97) (model 2), and for AHI, HOMA-IR, hsCRP, and oxG (OR: 0.94, 95% CI: 0.90–0.98) (model 3). In similar models adjusted for the same confounders, the presence of vitamin D deficiency was associated with 2–threefold greater odds of MetS (model 1: OR: 2.97, 95% CI: 1.50–5.85; model 2: OR: 3.02, 95% CI: 1.51–6.05; model 3: OR: 2.39, 95% CI: 1.15–4.97). The association between serum vitamin D status and MetS presence was mainly attributed to significant associations of both serum 25(OH)D levels (negative) and the presence of vitamin D deficiency (positive) with the likelihood of the lipidemic components of the MetS, i.e., hypertriglyceridemia and low levels of HDLC (Table 3). Results were similar in sensitivity analyses restricted among participants not receiving dietary supplements containing vitamin D (*n* = 241) (Online Resource 2).

**Table 3:** Multiple logistic regression analysis models exploring the association between serum vitamin D status and the presence of the metabolic syndrome and its components

	Serum 25(OH)D levels (per 1 ng/mL increase)			Vitamin D deficiency [25(OH)D < 20 ng/mL]		
Metabolic syndrome^a^	OR	95% CI	*P*	OR	95% CI	*P*
Model 1	0.926	0.887–0.968	0.001	2.967	1.504–5.851	0.002
Model 2	0.926	0.886–0.969	0.001	3.016	1.505–6.045	0.002
Model 3	0.939	0.897–0.984	0.008	2.388	1.148–4.968	0.020
Abdominal obesity^b^	OR	95% CI	*P*	OR	95% CI	*P*
Model 1	0.955	0.913–0.999	0.047	3.669	1.290–10.44	0.015
Model 2	0.962	0.917–1.008	0.104	3.256	1.126–9.413	0.029
Model 3	0.984	0.931–1.039	0.560	1.609	0.497–5.215	0.428
Hyperglycemia^c^	OR	95% CI	*P*	OR	95% CI	*P*
Model 1	0.936	0.886–0.990	0.020	1.856	0.838–4.109	0.127
Model 2	0.944	0.891–1.000	0.048	1.789	0.786–4.070	0.166
Model 3	0.961	0.903–1.023	0.210	1.360	0.531–3.487	0.522
Hypertriglyceridemia^d^	OR	95% CI	*P*	OR	95% CI	*P*
Model 1	0.941	0.900–0.984	0.010	2.513	1.271–4.969	0.008
Model 2	0.945	0.913–0.987	0.010	2.672	1.322–5.401	0.006
Model 3	0.950	0.920–0.992	0.015	2.564	1.230–5.348	0.012
Low HDLC^e^	OR	95% CI	*P*	OR	95% CI	*P*
Model 1	0.960	0.924–0.998	0.039	2.256	1.169–4.356	0.015
Model 2	0.959	0.922–0.999	0.043	2.324	1.183–4.564	0.014
Model 3	0.956	0.917–0.996	0.033	2.401	1.208–4.771	0.012
Hypertension^f^	OR	95% CI	*P*	OR	95% CI	*P*
Model 1	1.011	0.974–1.050	0.558	0.912	0.478–1.547	0.455
Model 2	1.012	0.974–1.052	0.542	0.896	0.471–1.559	0.453
Model 3	1.026	0.969–1.104	0.480	0.962	0.365–1.632	0.640

Model 1: adjusted for age, sex, and season of blood sample collection (winter–spring–summer–autumn). Model 2: adjusted for variables in Model 1 plus MedDietScore (range: 0–55), physical activity level (min/day), and smoking (never smokers–former smokers–current smokers). Model 3: adjusted for variables in model 2 plus AHI (events/h), HOMA-IR, hsCRP (mg/L), and oxG (ng/mg Cr).

AHI, apnea–hypopnea index; CI, confidence interval; DBP, diastolic blood pressure; HDLC, high-density lipoprotein cholesterol; HOMA-IR, homeostasis model of assessment of insulin resistance; hsCRP, high-sensitivity C reactive protein; MedDietScore, Mediterranean diet score; OR, odds ratio; oxG, oxidized guanine species; SBP, systolic blood pressure; TG, triglycerides; WC, waist circumference; 25(OH)D, 25-hydroxivitamin D.

^a^According to the criteria proposed by Alberti et al. [28]

^b^WC values > 102 cm for males and > 88 cm for females.

^c^Fasting glucose levels ≥ 100 mg/dL (≥ 5.6 mmol/L) or reception of antidiabetic medication.

^d^TG levels ≥ 150 mg/dL (≥ 1.7 mmol/L) or reception of lipid-lowering medication.

^e^HDLC levels < 40 mg/dL (< 1.0 mmol/L) for males and < 50 mg/dL (< 1.3 mmol/L) for females, or reception of relevant medication.

^f^SBP ≥ 130 mm Hg or/and DBP ≥ 85 mm Hg, or reception of antihypertensive medication.

## Discussion

Although OSA was traditionally viewed as an anatomical disease of the upper respiratory system, accumulated research over the past few decades has revealed that it actually represents a complex disease of cardiometabolic nature [33, 34]. Vitamin D status could mediate or explain the link between OSA and cardiometabolic morbidity, but research into this field is currently lacking. In the present cross-sectional study, we explored the relationship between serum 25(OH)D and the cardiometabolic profile of newly diagnosed patients with OSA, showcasing a significant correlation with anthropometric (BMI, WC), glucose metabolism (glucose, insulin, and HOMA-IR), lipidemic (TC, LDLC, HDLC, and TG), inflammatory (hsCRP), and oxidative stress (oxG) indices. Moreover, in multivariate analyses controlling for several confounders, both serum 25(OH)D levels and the prevalence of vitamin D deficiency emerged as significant predictors of the presence of MetS, indicating a strong independent link between serum vitamin D status and cardiometabolic health in patients with OSA.

Our findings revealed a high prevalence (63.0%) of vitamin D deficiency (< 20 ng/mL) among adult patients with OSA living in a typical Mediterranean country with many hours of sunlight. This observation might seem paradoxical but is in line with previous data showing high rates of vitamin D deficiency in various subgroups of the Greek population [35–37], as well as in Southern Europe and Eastern Mediterranean regions, in general [38]. Although the major source of vitamin D is skin synthesis after exposure to sunlight, a variety of factors could explain the observed high rates of vitamin D deficiency in our cohort [39, 40], namely, the following: obesity, which is highly prevalent among patients with OSA; higher skin melanin content; limited exposure to sunlight to avoid heat, especially during the summer; systematic use of sunscreens with high sun protection factor to comply with skin cancer prevention guidelines; limited outdoor physical activity, especially in urban environments (such as the metropolitan area of Athens); limited consumption of foods naturally rich in vitamin D (such as cod liver oil); limited consumption of foods fortified with vitamin D which are not abundant on the Greek market; and limited use of vitamin D supplements, as also supported by our findings (8.0%).

The favorable correlation of serum vitamin D status with cardiometabolic indices observed herein is in accord with the limited previously published epidemiological data in OSA populations [41–43]. For instance, in 2012, Bozkurt et al. [41] reported a significant negative association between serum 25(OH)D levels and glucose metabolism indices (fasting glucose, insulin, and HOMA-IR), as well as the prevalence of prediabetes and diabetes mellitus, assessed through a 75-g oral glucose tolerance test, among 143 patients with OSA. Moreover, the study of Barceló et al. [42] revealed negative correlations between serum 25(OH)D levels and fasting glucose, TC, and TG, as well as a significant age-, sex- and seasonality-adjusted trend of decreasing odds for diabetes mellitus and MetS with increasing serum 25(OH)D levels among 826 patients with OSA; when individual components of the MetS were analyzed, there were significant inverse associations of serum 25(OH)D levels with hypertriglyceridemia and low HDLC, but not with hyperglycemia and hypertension, similarly to our findings.

The inverse relationship of vitamin D status with MetS can be mediated, at least in part, by body weight status and body composition. In agreement with our findings, epidemiological data support an inverse association of serum vitamin D levels with body weight and other anthropometric indices related to visceral adiposity, such as WC. However, the direction and causality of the link between vitamin D deficiency and obesity or central obesity remains controversial, since weight loss leads only to minor improvements in vitamin D status, and the available interventional studies do not support a significant beneficial effect of vitamin D supplementation on body weight or composition [44, 45]. Multivariate analysis showed that the association between serum vitamin D status and MetS was mainly attributable to significant associations with the presence of hypertriglyceridemia and low levels of HDLC. It is possible that vitamin D has a significant impact on atherogenic dyslipidemia in OSA, which can be attributed to its ability to ameliorate insulin resistance and prevent increases in TG-rich lipoproteins, as well as to promote the formation of large HDL particles and enhance reverse cholesterol transport [46]. Moreover, there is a significant overlap in the metabolic pathways of vitamin D and cholesterol biosynthesis; for instance, 25(OH)D can suppress the activity of 3-hydroxy-3-methylglutaryl-CoA reductase, the rate-limiting enzyme for cholesterol synthesis, which is consistent with the observation that serum vitamin D is inversely associated with TC and LDLC, as also supported by our findings, and that vitamin D supplementation can reduce blood cholesterol [47]. The positive correlation between vitamin D and HDLC can be also partly attributed to the impact of vitamin D on HDL apolipoproteins; in interventional studies, an increase in apolipoprotein A1 levels has been observed after vitamin D supplementation, which might be the result of apolipoprotein A1 gene transcription enhancement in human hepatoma cells [48]. We also observed a significant negative correlation of serum 25(OH)D levels with plasma hsCRP, a standard marker of inflammation, and urinary oxG, a novel biomarker of DNA/RNA oxidative damage [49], which can indicate either the anti-inflammatory and anti-oxidant potential of vitamin D or the depletion of vitamin D stores in states of increased inflammation and oxidative stress, such as OSA [8, 9].

Collectively, our findings and those of previous epidemiological studies raise the possibility that reduced bioavailability and activity of 25(OH)D in OSA may facilitate the emergence and progression of the pathophysiology of the MetS. Moreover, in light of interventional data indicating that vitamin D supplementation may have beneficial effects on individual cardiometabolic risk factors (e.g., glucose metabolism, lipidemic profile, and inflammation) both in the general population [50] and in patients with OSA [51–53], vitamin D supplementation may prove beneficial for the management of both the respiratory and the cardiometabolic manifestations of OSA in vitamin D deficient subjects. Nevertheless, whether such an approach would lead to a meaningful reduction in cardiovascular risk in OSA remains unknown. According to the available well-designed randomized controlled clinical trials, vitamin D supplementation is not associated with reduced risk of major adverse cardiovascular events or lower cardiovascular mortality [54, 55], and, at the moment, routine use of vitamin D supplements is not recommended for the primary prevention of cardiovascular disease.

The present study is among the few that have assessed the relationship between serum 25(OH)D levels and the cardiometabolic profile of patients with OSA. The use of an attended overnight PSG for OSA diagnosis, the comprehensive assessment of patients’ cardiometabolic profile (including not only traditional biochemical indices but also sophisticated inflammatory and oxidative stress markers, and the presence of the MetS), the standardized protocol for serum 25(OH)D assays (all measurements were concomitantly performed using the same assays in the same laboratory, thereby reducing measurement variability), and the adequate confounding testing in statistical analyses are strong points of the present work. The major limitation of our study lies in its cross-sectional design, which does not allow exploration of causative associations and makes it impossible to infer whether vitamin D deficiency predisposes to MetS in OSA or vice versa. Additional limitations include the following: the fact that a single measurement of serum 25(OH)D may not reflect long-term vitamin D status; our study population consisting of adult Caucasian patients without significant comorbidities, which does not allow us to generalize the present findings in the whole OSA population; and the fact that participants’ lifestyle habits were assessed through questionnaires, which, despite being validated and appropriate for our study population, are prone to recall bias and, thus, misreporting cannot be excluded.

In conclusion, vitamin D deficiency is highly prevalent among adult newly-diagnosed patients with OSA and is associated with an adverse cardiometabolic risk profile. Given that both OSA and vitamin D deficiency are linked to cardiometabolic morbidity, efficient strategies to target both conditions are urgently needed. Future prospective epidemiological studies and randomized clinical trials should aim at evaluating whether vitamin D deficiency predisposes to MetS in patients with OSA or vice versa, and whether vitamin D supplementation can have a beneficial impact on both the respiratory and cardiometabolic features of OSA.

## Supplementary Information

Below is the link to the electronic supplementary material.

Supplementary file1 (DOCX 32 KB)

## Data Availability

Deidentified participant data will be made available by the corresponding author upon request for the purpose of data meta-analysis.
