# Supplementary material for: Associations between serum vitamin D status and the cardiometabolic profile of patients with obstructive sleep apnea
Source: Hormones (Athens). 2023 Jun 15;22(3):477–90. doi: 10.1007/s42000-023-00456-4 (PMC10449975; doi:10.1007/s42000-023-00456-4)
Supplement: Supplementary file 1 — Supplementary file1 (DOCX 32 KB) [file 42000_2023_456_MOESM1_ESM.docx]

**Online Resource 1.** Clinical and laboratory assessments of the study population.

**1.1 Anthropometric measurements**

Body weight (kg) was measured to the nearest 100 g with a digital scale (Tanita HD-351, Tokyo, Japan) placed on a hard-flat surface, with participants standing on the center of the scale without support, wearing light clothing and being barefoot, with their arms hanging loosely by their sides, their head facing forward, and their weight distributed evenly on both feet. Height (m) was measured to the nearest 0.5 cm with a stadiometer at the end of normal expiration, with participants’ weight equally distributed on their feet, with their head, upper back, buttocks, calves and heels on the vertical line of the stadiometer, and their head placed in the Frankfort horizontal plane (position of the head when the upper margin of the ear openings and lower margin of the orbit of the eye are horizontal). Based on body weight and height, the body mass index (BMI) was calculated as [$\mathrm{weight}\left( \mathrm{kg} \right)\div\mathrm{height}^{2} (m$)] and patients were categorized as underweight if BMI<18.5 kg/m^2^, normal-weight if 18.5≤BMI<25 kg/m^2^, overweight if 25≤BMI<30 kg/m^2^ or obese if BMI≥30 kg/m^2^ [Circulation. 2012;126(24):2870-7]. Waist circumference (WC) (cm) was measured to the nearest 0.1 cm between the lowest rib and the superior border of the iliac crest at the end of normal expiration, using a non-elastic measuring tape positioned parallel to the floor, and with the subject standing. All anthropometric measurements were performed by a trained and experienced dietitian.

**1.2 Laboratory analyses**

Fasting (12-h) blood samples and fist-morning urine samples were collected from participants following polysomnography. All biological samples were collected by experienced medical personnel, were centrifuged, and serum/plasma/urine samples were separated into 1.5 mL aliquots and frozen at -80 ◦C for future analyses. All aliquots were bar coded with a unique coded ID number for each participant. The following laboratory analyses were performed to measure biochemical, inflammatory and oxidative stress markers, as well as vitamin D.

1.2.1 Biochemical indices

Lipidemic profile indices, namely total cholesterol (TC) (mg/dL)^1^, high-density lipoprotein cholesterol (HDLC) (mg/dL)^1^ and triglycerides (TG) (mg/dL)^2^, were measured in plasma by an enzymatic colorimetric assay (COBAS® 8000 analyzer; F. Hoffmann-La Roche Ltd, Basel, Switzerland). Low-density lipoprotein cholesterol (LDLC) (mg/dL)^1^ was calculated using the Friedewald formula as [$\mathrm{TC}\left( md/dL \right)-HDLC (mg/dL)-(\frac{TG (mg/dL)}{5}$)]. Fasting glucose (mg/dL)^3^ was measured in plasma by an enzymatic colorimetric assay (COBAS® 8000 analyzer; F. Hoffmann-La Roche Ltd, Basel, Switzerland), insulin (μU/mL)^4^ was measured in plasma by chemiluminescence (E170 modular analyzer; F. Hoffmann-La Roche Ltd, Basel, Switzerland) and the homoeostasis model of assessment of insulin resistance (HOMA-IR) was calculated according to the formula of Matthews et al. as [$\frac{glucose (mg/dL) x insulin \left( \mu U/mL \right)}{450}$]. Liver enzymes, i.e., alanine transferase (ALT) (U/L), aspartate transferase (AST) (U/L) and gamma-glutamyl transpeptidase (GGT) (U/L), were measured in plasma by an enzymatic colorimetric method (COBAS® 8000 analyzer; F. Hoffmann-La Roche Ltd, Basel, Switzerland).

1 To convert cholesterol to mmol/L multiply mg/dL by 0.02586. 2 To convert triglycerides to mmol/L multiply mg/dL by 0.01129. 3 To convert glucose to mmol/L multiply mg/dL by 0.0555. 4 To convert insulin to pmol/L multiply μIU/L by 6.00.

1.2.2 Inflammatory and oxidative stress markers

High sensitivity CRP (hsCRP) (mg/L) levels were measured in plasma by nephelometry (nephelometric analyzer BN II System, Siemens Healthineers AG, Erlangen, Germany). Adiponectin levels (μg/mL) were measured in plasma by an immunoenzymatic method (Human Total Adiponectin Quantikine ELISA Kit, R&D Systems, Minneapolis, USA), with an intra-assay variation coefficient of <5% and an inter-assay variation coefficient of <8%. Urinary levels of 8-iso prostaglandin F2a (8-isoPGF2a) (ng/ml) were measured by an immunoenzymatic method (8-isoprostane ELISA Kit, Cayman Chemical, Michigan, USA), with an intra-assay variation coefficient of 7.2% and an inter-assay variation coefficient of 4.7%. Urinary oxidized guanine species (oxG) (ng/ml) were measured by an immunoenzymatic method (DNA/RNA Oxidative Damage ELISA Kit, Cayman Chemical, Michigan, USA), with an intra-assay variation coefficient of 15.5% and an inter-assay variation coefficient of 5.5%. Creatinine (Cr) levels (mg/mL) were measured in urine samples using the Jaffé method (Biosis Creatine Jaffé-Kinetics, Biotechnological Applications Ltd., Athens, Greece), based on which the urinary concentrations of 8-isoPGF2a and oxG were normalized and expressed as ng/mg Cr.

1.2.3 Vitamin D status

Total 25-hydroxyvitamin D [25(OH)D] (ng/mL)^5^ was measured in serum samples via chemiluminescence (LIAISON® 25 OH Vitamin D TOTAL Assay, Automated Analyzer Liaison Diasorin, ΜΑ-002/Α.8/04-04-2019, DiaSorin Inc., Stillwater, Minnesota, USA). The LIAISON® 25 OH Vitamin D assay is a direct, competitive chemiluminescent immunoassay (CLIA) for the quantitative determination of total 25(OH)D levels (25-hydroxyvitamin D2, 25-hydroxyvitamin D3, and other hydroxylated Vitamin D metabolites) in serum or plasma, with a measurement range of 4.0-150 ng/mL, an intra-assay coefficient of variation of 5.0% and an inter-assay coefficient of variation of 4.1%. In detail, during the first incubation, 25(OH)D is dissociated from its binding protein and binds to the specific antibody on the solid phase. After 10 minutes, the tracer (vitamin D linked to an isoluminol derivative) is added. After an additional 10-minute incubation, the unbound material is removed with a wash cycle. Subsequently, the starter reagents are added to initiate a flash chemiluminescent reaction. The light signal is measured by a photomultiplier as relative light units and is inversely proportional to the concentration of total 25(OH)D present in calibrators, controls, or samples.

5 To convert serum 25-hydroxyvitamin D to nmol/L multiply ng/mL by 2.5.

**1.3 Blood pressure measurement**

Blood pressure was measured with an automatic device operating on the oscillometric principle (OMRON HEM-7130, Kyoto, Japan). All measurements were performed in the left arm, after a 12-h fast and after at least a 30-min period without smoking or engaging in any kind of physical activity. The device was used to take two systolic (SBP) and diastolic blood pressure (DBP) measurements, separated by a 2-min interval, and their average was used for analyses.

**1.4 Presence of the metabolic syndrome**

The presence of the metabolic syndrome (MetS) was defined according to the 2009 joint criteria of the International Diabetes Federation Task Force on Epidemiology and Prevention, the National Heart, Lung, and Blood Institute, the American Heart Association, the World Heart Federation, the International Atherosclerosis Society, and the International Association for the Study of Obesity [Circulation. 2009;120(16):1640-5]. In specific, MetS was defined as the coexistence of ≥3 of the following components: (a) abdominal obesity, i.e., WC values >102 cm for males and >88 cm for females; (b) hyperglycemia, i.e., fasting glucose levels ≥100 mg/dL (≥5.6 mmol/L) but absence of a formal diagnosis of diabetes mellitus; (c) hypertriglyceridemia, i.e., TG levels ≥150 mg/dL (≥1.7 mmol/L) or reception of lipid-lowering medication; (d) low HDLC levels, i.e., <40 mg/dL (<1.0 mmol/L) for males and <50 mg/dL (<1.3 mmol/L) for females, or reception of relevant medication; and (e) hypertension, i.e., SBP ≥130 mm Hg or/and DBP ≥85 mm Hg, or reception of antihypertensive medication.

**Online Resource 2**. Multiple logistic regression analysis models exploring the association between serum vitamin D status and the presence of the metabolic syndrome and its components among participants not receiving dietary supplements containing vitamin D (n=241).

|  | **Serum 25(OH)D levels (per 1 ng/mL increase)** | | | **Vitamin D deficiency [25(OH)D <20 ng/mL]** | | |
| --- | --- | --- | --- | --- | --- | --- |
| **Metabolic syndrome ^a^** | **OR** | **95% CI** | **P** | **OR** | **95% CI** | **P** |
| Model 1 | 0.933 | 0.892-0.977 | 0.003 | 2.664 | 1.309-5.425 | 0.007 |
| Model 2 | 0.934 | 0.891-0.979 | 0.004 | 2.707 | 1.301-5.635 | 0.008 |
| Model 3 | 0.945 | 0.900-0.991 | 0.020 | 2.283 | 1.054-4.944 | 0.036 |
| **Abdominal obesity ^b^** | **OR** | **95% CI** | **P** | **OR** | **95% CI** | **P** |
| Model 1 | 0.959 | 0.914-1.005 | 0.082 | 3.062 | 0.979-9.584 | 0.055 |
| Model 2 | 0.964 | 0.918-1.013 | 0.151 | 2.732 | 0.855-8.737 | 0.090 |
| Model 3 | 0.986 | 0.931-1.043 | 0.617 | 1.399 | 0.398-4.924 | 0.601 |
| **Hyperglycemia ^c^** | **OR** | **95% CI** | **P** | **OR** | **95% CI** | **P** |
| Model 1 | 0.950 | 0.898-1.006 | 0.079 | 1.743 | 0.760-3.998 | 0.189 |
| Model 2 | 0.959 | 0.904-1.017 | 0.161 | 1.676 | 0.701-4.007 | 0.245 |
| Model 3 | 0.979 | 0.920-1.043 | 0.518 | 1.208 | 0.447-3.267 | 0.710 |
| **Hypertriglyceridemia ^d^** | **OR** | **95% CI** | **P** | **OR** | **95% CI** | **P** |
| Model 1 | 0.955 | 0.925-0.994 | 0.015 | 2.137 | 1.055-4.327 | 0.035 |
| Model 2 | 0.959 | 0.926-0.995 | 0.020 | 2.264 | 1.092-4.696 | 0.028 |
| Model 3 | 0.962 | 0.927-0.999 | 0.048 | 2.313 | 1.074-4.978 | 0.032 |
| **Low HDLC ^e^** | **OR** | **95% CI** | **P** | **OR** | **95% CI** | **P** |
| Model 1 | 0.964 | 0.926-0.998 | 0.030 | 2.110 | 1.057-4.213 | 0.034 |
| Model 2 | 0.960 | 0.922-0.998 | 0.023 | 2.049 | 1.006-4.171 | 0.048 |
| Model 3 | 0.966 | 0.927-1.000 | 0.050 | 2.092 | 1.014-4.316 | 0.046 |
| **Hypertension ^f^** | **OR** | **95% CI** | **P** | **OR** | **95% CI** | **P** |
| Model 1 | 1.005 | 0.967-1.045 | 0.790 | 1.006 | 0.473-2.142 | 0.987 |
| Model 2 | 1.002 | 0.963-1.044 | 0.906 | 1.074 | 0.493-2.337 | 0.858 |
| Model 3 | 1.015 | 0.971-1.060 | 0.518 | 0.995 | 0.354-2.220 | 0.599 |
| Model 1: adjusted for age, sex and season of blood sample collection (Winter – Spring – Summer – Autumn). Model 2: adjusted for variables in Model 1 plus MedDietScore (range: 0-55), physical activity level (min/d) and smoking (never smokers – former smokers – current smokers). Model 3: adjusted for variables in Model 2 plus AHI (events/hour), HOMA-IR, hsCRP (mg/L) and oxG (ng/mg Cr).  ^a^ According to the criteria proposed by Alberti et al. [Circulation. 2009;120(16):1640-5]. ^b^ WC values >102 cm for males and >88 cm for females. ^c^ Fasting glucose levels ≥100 mg/dL (≥5.6 mmol/L) or reception of antidiabetic medication. ^d^ TG levels ≥150 mg/dL (≥1.7 mmol/L) or reception of lipid-lowering medication. ^e^ HDLC levels <40 mg/dL (<1.0 mmol/L) for males and <50 mg/dL (<1.3 mmol/L) for females, or reception of relevant medication. ^f^ SBP ≥130 mm Hg or/and DBP ≥85 mm Hg, or reception of antihypertensive medication.  Abbreviations: AHI, apnea-hypopnea index; CI, confidence interval; DBP, diastolic blood pressure; HDLC, high-density lipoprotein cholesterol; HOMA-IR, homeostasis model of assessment of insulin resistance; hsCRP, high-sensitivity C reactive protein; MedDietScore, Mediterranean diet score; OR, odds ratio; oxG, oxidized guanine species; SBP, systolic blood pressure; TG, triglycerides; WC, waist circumference; 25(OH)D, 25-hydroxivitamin D. | | | | | | |
